# Routine Postsurgical Anesthesia Visit to Improve 30-day Morbidity and Mortality: A Multicenter, Stepped-wedge Cluster Randomized Interventional Study (The TRACE Study)

**DOI:** 10.1097/SLA.0000000000004954

**Published:** 2021-05-24

**Authors:** The TRACE Study Investigators

**Keywords:** anesthesiology, failure to rescue, postoperative complications, postoperative mortality

## Abstract

To study the impact of a standardized postoperative anesthesia visit on 30-day mortality in medium to high-risk elective surgical patients.

**Background:**

Postoperative complications are the leading cause of perioperative morbidity and mortality. Although modified early warning scores (MEWS) were instituted to monitor vital functions and improve postoperative outcome, we hypothesized that complementary anesthesia expertise is needed to adequately identify early deterioration.

**Methods:**

In a prospective, multicenter, stepped-wedge cluster randomized interventional study in 9 academic and nonacademic hospitals in the Netherlands, we studied the impact of adding standardized postoperative anesthesia visits on day 1 and 3 to routine use of MEWS in 5473 patients undergoing elective noncardiac surgery. Primary outcome was 30-day mortality. Secondary outcomes included: incidence of postoperative complications, length of hospital stay, and intensive care unit admission.

**Results:**

Patients were enrolled between October 2016 and August 2018. Informed consent was obtained from 5473 patients of which 5190 were eligible for statistical analyses, 2490 in the control and 2700 in the intervention group. Thirty-day mortality was 0.56% (n = 14) in the control and 0.44% (n = 12) in the intervention group (odds ratio 0.74, 95% Confidence interval 0.34–1.62). Incidence of postoperative complications did not differ between groups except for renal complications which was higher in the control group (1.7% (n = 41) vs 1.0% (n = 27), *P* = 0.014). Median length of hospital stay did not differ significantly between groups. During the postanesthesia visits, for 16% (n = 437) and 11% (n = 293) of patients recommendations were given on day 1 and 3, respectively, of which 67% (n = 293) and 69% (n = 202) were followed up.

**Conclusions:**

The combination of MEWS and a postoperative anesthesia visit did not reduce 30-day mortality. Whether a postoperative anesthesia visit with strong adherence to the recommendations provided and in a high-risk population might have a stronger impact on postoperative mortality remains to be determined.

**Trial Registration:**

Netherlands Trial Registration, NTR5506/ NL5249, https://www.trialregister.nl/trial/5249.

Postoperative complications are the leading cause of perioperative morbidity and mortality.^1,2^ In 2010, the overall mortality of surgical procedures in the Netherlands was 1.85%.^3^ Delayed recognition and treatment of deteriorating patients on the ward contributes to severe postoperative complications and mortality,^4,5^ also addressed as failure-to-rescue.^6^

To reduce postoperative mortality in the Netherlands, a bundle of measures including modified early warning scores (MEWS) and rapid response teams were introduced. However, these measures were not specifically developed and validated for the perioperative setting. Postoperatively, patient deterioration starts with mild abnormalities of vital signs. The sensitivity of the MEWS alone is insufficient to detect this early phase of deterioration and needs to be combined with medical expertise.^7^

Anesthesiologists are trained to identify deterioration of vital functions in an early phase. However, the anesthesiologists expertise is not structurally embedded in the perioperative process outside the operating room. Therefore, a standardized postoperative anesthesia visit may improve early recognition of deterioration and reduce failure to rescue. Currently, studies assessing the impact of such an intervention on postoperative mortality are scarce.

The Routine posTsuRgical Anesthesia visit to improve patient outComE (TRACE) study assessed the impact of combining the MEWS with a standardized postoperative anesthesia visit on patient outcome. We hypothesized that routine postsurgical anesthesia visits reduce 30-day morbidity and mortality in adult patients undergoing noncardiac surgery.

## Methods

### Study Design

TRACE was a prospective, multicenter, stepped-wedge cluster randomized interventional study in patients who underwent noncar-diac surgery in 9 academic and nonacademic hospitals in the Netherlands. The TRACE population reflects a representative sample of in-hospital patients undergoing moderate to high risk surgery. A full description of the methods of the TRACE study has been published.^8^ (Link to online study protocol: https://www.ncbi.nlm.-nih.gov/pmc/articles/PMC6204052/)

Ethical approval was obtained from the Human Subjects Committee of Amsterdam UMC, location VUmc Amsterdam (number NL56004.029.16). The study was registered in the Netherlands Trial Register (NTR5506). Patient inclusion and data registration was monitored by the Clinical Research Unit of the Amsterdam UMC.

### Participants

Eligible patients underwent elective noncardiac surgery and had an indication for postoperative hospital stay. Patients met 1 or more of the following criteria: age ≥60 years; age ≥45 years with a revised cardiac risk index >2; age ≥ 18 years with an indication for postoperative invasive pain therapy; age ≥18years with a postoperative surgical APGAR-score <5. Patients were only included in the study after written informed consent was obtained. Participants were recruited by a member of the study team during the preoperative screening or preoperative hospital admission. Patients with an indication for a postoperative stay in the intensive care unit (ICU) were excluded from study participation.

### Randomization

According to the stepped-wedge design, at the start of the study all participating hospitals provided standard perioperative care. This was followed by a stepwise introduction of the intervention. The order in which the hospitals started with the intervention was randomized using envelope drawing.^8^ During the recruitment phase, 1 of the initial 8 hospitals dropped out of the study and a ninth hospital was added.

### Procedures

Standard postoperative care on the normal ward was provided by the surgical team. According to national guidelines, patients are monitored by the surgical ward team (ward nurses and a ward physician), including determination of the MEWS score at least 3 times a day. A staff surgeon visits the ward patients at least once a day during a ward round with the ward nurse and ward physician.

The intervention added routine visits by an anesthesiologist on postoperative days 1 and 3 to standard care. In case of (suspected) deterioration of the patient’s condition, the anesthesiologist advised the treating physician on further diagnostics, treatment, and follow-up of the patient. This recommendation was documented in the electronic medical record.

### Outcome Measures

The primary outcome measure was postoperative 30-day mortality. Secondary outcome measures were the incidence of postoperative complications (for definitions, see Appendix 1, http://links.lww.com/SLA/D148), length of stay in the hospital, incidence and length of stay in the ICU, and quality of life 7 and 30 days after surgery. Follow-up of the anesthesiologist’s recommendation by the treating physician was recorded additionally.

### Statistical Analyses

Based on previous data from the Netherlands we estimated the incidence of postoperative mortality to be 2%.^3^ A difference in mortality of 50% between the control and intervention group was regarded as clinically relevant. To detect a reduction from 2% to 1% in postoperative 30-day mortality, with an alpha of 0.05 and a power of 80%, and a 1:1 ratio between the control and the intervention group, a total of 4638 patients had to be included in a parallel randomized controlled trial design. To compensate for inter-institutional variation, a possible time-effect inherent to the stepped-wedge design, and drop-out, we increased this sample size with 20%, resulting in an estimated sample size of 5600 patients. Patient characteristics were described using mean and standard deviation (SD) for continuous variables, and count and percentage for categorical variables. The primary outcome, 30-day mortality, was compared between groups using logistic mixed-effects regression analysis with a random intercept and slope. Group differences were adjusted for time effects and baseline characteristics that differed between groups to a clinically meaningful extent. If necessary, time was modeled using restricted cubic splines to deviate from the linearity assumption. The Akaike Information Criterion was used to determine the best fitting model. Given the study design, additional correction for type 1 error was not necessary.

Secondary outcomes were tested between groups using either linear or logistic mixed-effects regression with a link-function depending on the distribution of the outcome, with a similar random effects structure as for the primary outcome measure.

Analyses were performed according to the intention to treat principle and per protocol. The per protocol analysis included only those patients hospitalized until at least postoperative day 3, and in the intervention group those patients who actually received the postsurgical visits according to protocol. In case of missing data, an appropriate imputation method according to the nature of miss-ingness was selected.

### Role of the Funding Source

The funder of the study had no role in study design, data collection, data analysis, data interpretation, or writing of the report. The corresponding author had full access to all study data and took final responsibility for the decision to submit for publication.

## RESULTS

**Figure 1:**
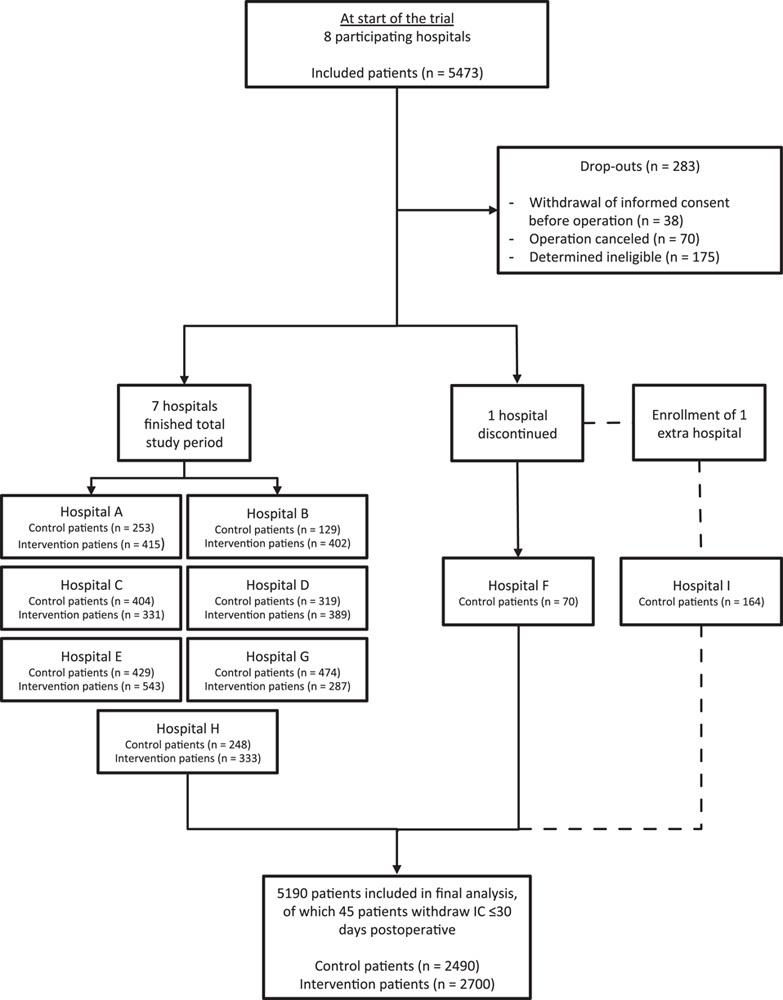
Patient flow diagram.

This study was done and reported in accordance with CONSORT guidelines for stepped wedge cluster randomized trials.^9^ Patients were enrolled between October 2016 and August 2018. The willingness to participate in the study was high, and the main reported reason to not participate in the study was the burden of completing the questionnaires. In total, informed consent was obtained from 5473 patients of which 5190 were eligible for statistical analyses. The control group consisted of 2490 patients and 2700 patients were allocated to the intervention group (Fig. 1). Patient characteristics and types of surgery were balanced between groups, except for clinically meaningful differences in the prevalence of active cancer and renal disease (Table 1). Therefore, all further analyses were adjusted for active cancer and renal disease.

**Table 1:** Baseline characteristics

	Control Period (n = 2490)	Intervention Period (n = 2700)
Mean age (yr)	67 (61 – 73)	67 (61 – 73)
Women	1169 (47)	1300 (48)
Mean BMI (kg/m^2^)	27 (23.5 – 30.5)	27 (23.5 – 30.5)
Activity level (<4 METs)	151 (6.1)	155 (6)
ASA classification		
I	231 (9)	232 (9)
II	1536 (62)	1627 (60)
III	697 (28)	803 (30)
IV	23 (1)	37 (1)
Revised cardiac risk index (rCRI)		
0 points	1020 (41)	1194 (44)
1 point	1151 (46)	1161 (43)
2 points	250 (10)	279 (10)
3 points	50 (2)	46 (2)
4 points	9 (0)	8 (0)
5 points	1 (0)	0 (0)
Comorbid disorders*		
Active cancer	945 (38)	1153 (43)
Cardiovascular disease	654 (26)	706 (26)
Cerebrovascular disease	181 (7)	196 (7)
Diabetes mellitus	381 (15)	411 (15)
Pulmonary disease	256 (10)	283 (10)
Renal disease	190 (7)	351 (13)
Type of surgery†		
Ear, nose and throat	76 (3)	96 (3)
Gastrointestinal or liver	749 (30)	902 (33)
Gynaecological	204 (8)	204 (7)
Orthopaedic, arthroplasty and spine	479 (19)	473 (17)
Thoracic	108 (4)	117 (4)
Urological	442 (17)	457 (17)
Vascular	168 (7)	207 (8)
Other	348 (14)	289 (11)
Grade of surgery		
High risk surgery‡	1085 (44)	1105 (41)
Quality of life status		
EQ5D5L utility (mean, SD)	0.78 (0.23)	0.79 (0.22)

Data are median (IQR) and n (%), unless otherwise stated.

* For definitions see Appendix 1A, http://links.lww.com/SLA/D148.

† Some patients may have had more than 1 type of surgery; for definitions see Appendix 1B, http://links.lww.com/SLA/D148.

‡ Intraperitoneal, intrathoracic, or suprainguinal vascular surgery, according to rCRI.

ASA indicates American Society of Anesthesiologists; BMI, body mass index; EQ-5D-5L, EuroQol 5- dimension 5-level; MET, metabolic equivalence of task; rCRI, revised cardiac risk index; SD, standard deviation.

The primary outcome was recorded for all patients. Fourteen patients (0.56%) in the control group died within 30days after surgery compared to 12 patients (0.44%) in the intervention group.

The odds ratio for 30-day mortality was 0.74 (95% confidence interval: 0.34–1.62; *P* = 0.45). Although overall mortality in patients with cancer at baseline was similar in both groups (1.1% in the control, 1.0% in the intervention group), 10 out of 14 (71.4%) in the control versus 11 out of 12 (91.7%) in the intervention group died with cancer.

Twenty-three out of 26 patients died in the hospital. Eleven (0.41%) in the intervention and 12 (0.48%) in the control group and 3 (0.06%) patients died after hospital discharge. Of the 23 in-hospital deaths, 12 fulfilled the criteria of failure to rescue (mortality during index hospitalization following postoperative complications).

The incidence of any renal complication was higher in the control group (1.7%) than in the intervention group (1.0%; *P* = 0.014). All other complications did not differ between groups (Table 2).

**Table 2:** Postoperative complications

	Total N = 5190	Control N = 2490	Intervention N = 2700	P-value N = 5190
Postoperative complications				
Infectious complications, any	385 (7.4%)	166 (6.7%)	219 (8.1%)	0.386
SSI, any	183 (3.5%)	83 (3.3%)	100 (3.7%)	0.993
Superficial surgical site	134 (2.6%)	64 (2.6%)	70 (2.6%)	
Deep surgical site	22 (0.4%)	9 (0.4%)	13 (0.5%)	
Organ space SSI	38 (0.7%)	16 (0.6%)	22 (0.8%)	
Pneumonia	150 (2.9%)	62 (2.5%)	88 (3.3%)	
Urinary tract infection	87 (1.7%)	35 (1.4%)	52 (1.9%)	
Sepsis, Septic shock	51 (1.0%)	23 (0.9%)	28 (1.0%)	
Cardiac complications, any	179 (3.4%)	75 (3.0%)	104 (3.9%)	0.106
Myocardial infarction	8 (0.2%)	5 (0.2%)	3 (0.1%)	
Cardiac arrest	9 (0.2%)	8 (0.3%)	1 (0.0%)	
Heart failure	20 (0.4%)	4 (0.2%)	16 (0.6%)	
Arrhythmia	152 (2.9%)	65 (2.6%)	87 (3.2%)	
Pulmonary complications, any	134 (2.6%)	59 (2.4%)	75 (2.8%)	0.674
On ventilator 48 h	60 (1.2%)	26 (1.1%)	34 (1.3%)	
Unplanned re-intubation	25 (0.5%)	13 (0.5%)	12 (0.5%)	
Pulmonary oedema	70 (1.3%)	29 (1.2%)	41 (1.5%)	
Thromboembolic and vascular complications, any	53 (1.0%)	26 (1.0%)	27 (1.0%)	0.760
Deep venous thrombosis	9 (0.2%)	3 (0.1%)	6 (0.2%)	
Pulmonary embolism	27 (0.5%)	16 (0.6%)	11 (0.4%)	
Stroke, CVA	17 (0.3%)	7 (0.3%)	10 (0.4%)	
Renal complication, any	68 (1.3%)	41 (1.7%)	27 (1.0%)	0.014
Acute renal failure	9 (0.2%)	5 (0.2%)	4 (0.2%)	
Progressive renal insufficiency	59 (1.1%)	36 (1.5%)	23 (0.9%)	
Surgical complications, any	145 (2.8%)	60 (2.4%)	85 (3.2%)	0.191
Anastomotic leakage	89 (1.7%)	39 (1.6%)	50 (1.9%)	
Postoperative bleed	63 (1.2%)	23 (0.9%)	40 (1.5%)	
Reoperation	157 (3.0%)	81 (3.3%)	76 (2.8%)	0.337
Other complications, any	888 (17.1%)	419 (16.8%)	469 (17.4%)	0.275
Allergic reaction	16 (0.3%)	7 (0.3%)	9 (0.3%)	
Delirium	104 (2.0%)	50 (2.0%)	54 (2.0%)	
Ileus	122 (2.3%)	56 (2.3%)	66 (2.5%)	
Other	813 (15.7%)	387 (15.5%)	426 (15.8%)	

Postoperative complications for 5190 patients undergoing elective surgery.

Some patients may have developed more than 1 complication.

CVA indicates cerebrovascular accident; SSI, surgical site infection.

Median length of hospital stay was 4 days (range 0–112) in the control group and 4 days (range 1 – 130) in the intervention group (*P* = 0.31). One hundred patients (4.0%) in the control group compared to 89 patients (3.3%) in the intervention group (*P* = 0.20) were admitted to the ICU in the postoperative period. The median length of stay in the ICU was 2 days (range 1 – 70) and 2 days (range 166) in the control and intervention group, respectively (*P* = 0.45). In total, 43 patients in the control group were admitted to a medium care facility (1.7%), compared to 67 (2.5%) in the intervention group (*P* = 0.26). The median length of stay was 1 day (range 1–24) in the control group compared to 2 days (range 1 – 36) in the intervention group.

The average (SD) quality of life score on the seventh day after surgery was 0.73 (0.22) in the intervention group compared to 0.72 (0.23) in the control group (*P* = 0.92). At 30 days, the average (SD) quality of life score was 0.80 (0.19) in both groups (*P* = 0.10).

A postoperative recommendation by the anesthesiologist was provided for 437 patients (16%) on day 1 and 293 patients (11%) on day 3. This advice was followed by the treating physician in 67% of the cases on day 1 and 69% of the cases on day 3. The majority of recommendations consisted of optimization of pain therapy (28.0% of the provided advices on day 1 and 23.2% on day 3), adjustment of medication (15.4% and 16.3%) or adherence to elements of the enhanced recovery after surgery guidelines, (40.2% and 41.7%).

In total, 1176 patients (47.2%) in the control group were discharged before the third day compared to 1167 patients (43.2%) in the intervention group (*P* = 0.238). In the intervention group, 330 patients (12.2%) did not receive all planned visits by the anesthesiologist. In total, 1176 patients from the control group were omitted from the per protocol analysis, and 1392 from the intervention group. Hence, 1314 remained in the control group, 1308 in the intervention group. Of those, 10 in each group died within 30 days after surgery (ie, 0.76% in the control group and 0.76 in the intervention group, *P* = 0.88). Thus, results from the per protocol analysis were similar to the intention to treat analysis.

## Discussion

In our study involving 5190 patients who underwent inpatient noncardiac surgery in 9 hospitals in the Netherlands, 26 (0.5%) died within 30 days after surgery, of which 23 died in the hospital.

The combination of MEWS and a postoperative anesthesia visit did not reduce the primary outcome 30-day mortality. All secondary outcome parameters were not different between the control and the intervention group, except for any renal complication, which was significantly lower in patients in the intervention group.

TRACE is the first prospective interventional study assessing the impact of a structured postoperative anesthesia visit on postoperative outcome. With the use of the revised cardiac risk index and the surgical APGAR-score we aimed to include patients with an intermediate to high perioperative risk profile by taking into account both patient- and surgical-specific factors. Although this resulted in a comparable patient population compared with other recent studies, mortality in TRACE is considerably lower than in most of these studies (0.4% to 5.8%).^10–14^ Failure to rescue occurred in only 12 patients (0.23%), which is also lower than reported in previous studies.

In addition, we found a low incidence of postoperative complications. Cardiovascular complications occurred in 3.4% of patients, sepsis in 1%, postoperative bleeding in 1.2%, and renal complications in 1.3%. In contrast, within the VISION cohort the incidence of cardiovascular complication, sepsis, and major bleeding was 19.4%, 4.1%, and 14.2%, respectively, all 3 associated with postoperative mortality.^11^ This difference may explain the lower mortality in TRACE.

In the Netherlands, perioperative mortality in intermediate risk patients was 1.85% in 2010^3^ and 2.0% in 2012.^10^ To improve patient outcome and to reduce failure to rescue, a nationwide surgical safety program was implemented in 2010. This program consisted of measures to prevent the occurrence of perioperative complications (safe surgery guidelines, postoperative wound infection bundle, standardized infection prevention) and measures to earlier detect and treat any complication in the postoperative period (MEWS, rapid response teams, acute pain service). Widespread implementation of less invasive surgical procedures in the last decade potentially further reduced the impact of surgical risk on patient outcome. In addition, prehabilitation programs, in particular for patients undergoing cancer and orthopedic surgery were introduced.^15^

The observed mortality rate in TRACE suggests that the abovementioned measures have led to improvement in standard of care in recent years. Given the low incidence of complications in TRACE, our intervention as a measure to prevent failure to rescue was less likely to result in a significant reduction in mortality.

Postoperative complications for 5190 patients undergoing elective surgery. Some patients may have developed more than 1 complication.

CVA indicates cerebrovascular accident; SSI, surgical site infection.

Our findings with respect to the primary outcome do not support the hypothesis of the trial. However, we cannot ignore the potential effect of cancer-related death in the TRACE study. Although 30-day postoperative mortality in patients with cancer is similar in the control versus the intervention group (1.1% in the control group and 1.0% in the intervention group), the proportion of patients with cancer-related death is higher in the intervention group. The imbalance in cancer-related death in the intervention group might have reduced the impact of the postoperative anesthesia visit, although even after correction for baseline imbalances we still did not find a difference in mortality.

Furthermore, the anesthesiologist provided a substantial amount of medical advice to the ward staff and the treating physician, suggestive of potential for improvement in postoperative care. Those recommendations for improving care pertained to optimization of pain therapy in about one-fifth of the cases, means to improve oxygenation, fluid therapy, infection control, and in about 40% additional diagnostic measures or adherence to elements of the enhanced recovery after surgery guidelines. It should be emphasized that one-third of those recommendations were only partially or not followed up by the treating physician. Whether this lack of compliance has contributed to the lack in difference in our primary outcome remains speculative.

Our study has several limitations. First, we did not reach the number of 5600 patient inclusions because 1 hospital stopped participation for logistic reasons. This hospital was replaced by another hospital during the recruitment period. Nonetheless, the TRACE study group achieved recruitment of 5473 patients within 1 year. Because the drop-out rate was only 5%, and we anticipated a 20% correction combined for drop-out and cluster- and time-effects, our study is still sufficiently powered.

Second, all postoperative visits were performed by anesthesiologists or residents with at least 1-year experience in the ICU. Inter-individual differences may have occurred in the approach of the visits, but by offering standardized instructions we ensured to keep this to a minimum.

To increase the success of an intervention that further reduces 30-day postoperative mortality, by lowering failure to rescue, future research should focus on a well-defined high-risk patient population and organizational measures to embed the postoperative anesthesia visit within the regular surgical rounds, facilitating follow-up of recommendations provided. In conclusion, the combination of MEWS and a postoperative anesthesia visit did not reduce 30-day mortality. Given the observed low incidence of postoperative complications and consequently overall mortality in TRACE, it turned out to be difficult to prove effectiveness of interventions aiming at reducing failure to rescue in the Dutch health care system. Whether a postoperative anesthesia visit with strong adherence to the recommendations provided and in a high-risk population might have a stronger impact on postoperative complications and mortality remains to be determined.
